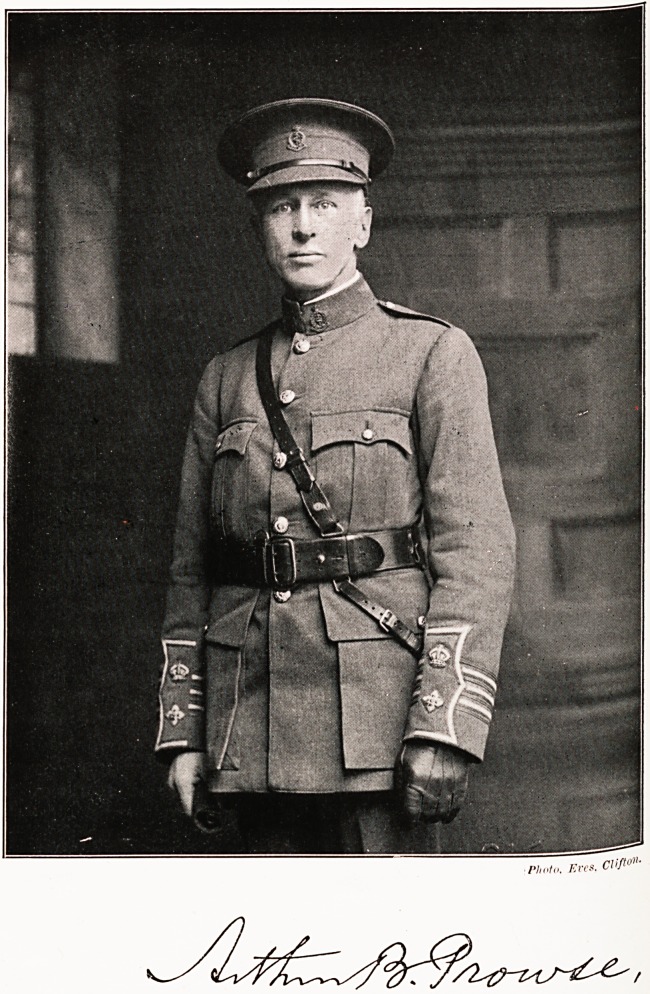# Arthur Bancks Prowse

**Published:** 1925

**Authors:** 


Arthur B. Prowse.

### ARTHUR BANCKS PROWSE, M.D., F.R.C.S.

Arthur Bancks Prowse, whose death after a long illness bravely and patiently borne we all deeply regret, was born on March 27th, 1856, at Millbrook, near Plymouth. He was the eldest son of Dr. Wm. Prowse and grandson of Dr. James Prowse, who practised for many years in St. James' Barton. His early education was received at Amersham Grammar School and Amersham Hall. His medical training began at Liverpool and was completed at St. Mary's Hospital, London. In 1877 he took his M.R.C.S. and in the following year his M.B. London, with honours in all subjects. In 1881 he became M.D. and in 1882 F.R.C.S. He filled the posts of House Surgeon to the Westminster Ophthalmic Hospital and the London Fever Hospital and was House Physician and Medical Registrar at St. Mary's Hospital, where he also obtained a scholarship in Anatomy. Early settling in Bristol, he was appointed Assistant Physician at the Royal Infirmary in 1883, and continued on the staff for many years as Physician, Dean of the Medical Faculty and Lecturer in Materia Medica, Pharmacology and Therapeutics. He also acted as Hon. Physician to the Blind Women's Home, Clifton, the Clifton Crêche and the Blue Maids' Orphanage, Ashley Hill. When the British Medica Association met in Bristol in 1895 his services as Hon. Financial Secretary were invaluable.

At the outbreak of the War he was an *à la suite* officer of the 2nd Southern General Hospital. Colonel Bush writes of his work: "His sound judgment and the great attention he gave to details very soon proved him to be a most methodical officer, his services being utilised as a physician in the wards, and as a member or president of a vast number of Medical Boards. He was also placed in charge of the arrangements for moving the sick and wounded soldiers to and from the auxiliary hospitals attached to the 2nd Southern. In June, 1917, when Colonel Paul Bush was called to do duty in France, the command of that establishment was conferred on him with the rank of Lieut.-Colonel. He remained as administrator in command until the hospital was closed on April 22nd, 1919. His work and his personality endeared him to the many thousand patients who passed through his hands during the Great War. The officers, non-commissioned officers and men of his unit looked upon him as a very able administrator and a good friend."

In addition to his purely professional duties he was a man of varied activities and many hobbies. He was never idle, and when at last confined to his bed he was surrounded with books and papers, and as long as his hand could hold a pen was busy with one or other of his favourite pursuits. It was said by an old Spanish physician that if you know only medicine you don't know that, and no one followed this advice more than he. A great lover of Devonshire, and knowing almost every inch of ground on Dartmoor, and deeply interested in its antiquities, he compiled from the fifty-six volumes of the Devonshire Association for the Advancement of Science, Literature and Art a complete index of place-names—a work which involved an immense amount of labour and patience—and up to the time of his death he was preparing a card index on the origin of all the place-names in Devon for the English Place-Names Society. To local antiquarian research he devoted much time, contributing two papers to the Bristol Naturalists' Society's *Transactions* on "Some Ancient British Remains on Clifton Down," and "Some Ancient British Remains near Long Ashton, Somerset"; but perhaps the most important work he did in this direction was when some years ago he succeeded in tracing the long-lost course of Wansdyke from Maes Knoll to the foot of Dundry Hill. In a letter written by Mr. Albany F. Major, and lately published in the *Bristol Times and Mirror*, he says: "He read an account of this to the local society, but curiously enough the importance of this discovery was not recognised and the paper was never printed. Nothing was known of it outside his immediate circle till in 1923, on one of my visits to Somerset to investigate the course of Wansdyke, I fortunately heard of it and got into touch with Dr. Prowse. He most kindly placed all his knowledge at my disposal, took me over the ground, and joined me in further investigations in that and the following year. It was my great pleasure to be able to bring his discovery to the notice of the Somersetshire Archæological and Natural History Society." Dr. Prowse was long associated with the Bristol Naturalists' Society, of which he was formerly a President, and for the great interest he took in it and for his long and valuable services as Librarian he was elected an Honorary Member. He was most methodical in all he did, sparing no labour, and his work was characterised by extreme neatness and accuracy. He was a man of pronounced and very strong opinions, and although they often failed to coincide with those of his friends they were never based on the mere *ipse dixit* of anyone, but were the result of most painstaking research and deep knowledge. Compromise was alien to his nature, and his somewhat reserved manner debarred those who did not know him from realising the beauty of his character. Deeply religious, his religion seemed to be bound up with his love of flowers, and he must have experienced the same feeling that Brown, the Manx poet, had:

> "Not God; in gardens! when the eve is cool?
> Nay, but I have a sign!
> 'Tis very sure God walks in mine."

One of his prospective joys was when he could live elsewhere and have a garden of his own. He made the most, however, of the little patch in Lansdown Place, utilising every inch and growing many rare and beautiful plants. But his long connection with the Parish Church, Clifton (of which he was warden for twenty-seven years and for which he worked so hard, keeping the accounts in his usual careful manner and saving the parishioners much expense by his personal labours), enabled him to have the churchyard under his care. He found it a wilderness: he left it one of the beauty spots of Clifton. His memory will always be associated with this happy record of his skill and love of flowers. Many are the rare and fine flowering shrubs he obtained for it, and it was on his initiative that the trees lining the walk from Victoria Square to Clifton Hill were replenished and trained to form the beautiful avenue it is. Bacon said: "God Almighty first planted a garden; and indeed it is the purest of humane pleasure." His love of flowers and love of music reflected his character, and it may be said of him as of Sir Galahad:—

> "His strength was of the strength of ten
> Because his heart was pure."

## Figures and Tables

**Figure f1:**